# High Fat Diet-Induced Hepatic 18-Carbon Fatty Acids Accumulation Up-Regulates CYP2A5/CYP2A6 via NF-E2-Related Factor 2

**DOI:** 10.3389/fphar.2017.00233

**Published:** 2017-05-15

**Authors:** Xing-he Wang, Xiao-xu Cui, Xiao-qi Sun, Xing-hui Wang, Xiao-chong Li, Yue Qi, Wei Li, Mei-yu Han, Ishfaq Muhammad, Xiu-ying Zhang

**Affiliations:** Laboratory of Veterinary Pharmacology and Toxicology, Faculty of Basic Veterinary Science, College of Veterinary Medicine, Northeast Agricultural UniversityHarbin, China

**Keywords:** 18-carbon fatty acid, CYP2A5, CYP2A6, Nrf2, high-fat diet, hepatocyte steatosis

## Abstract

To investigate the role of hepatic 18-carbon fatty acids (FA) accumulation in regulating CYP2A5/2A6 and the significance of Nrf2 in the process during hepatocytes steatosis, Nrf2-null, and wild type mice fed with high-fat diet (HFD), and Nrf2 silenced or over expressed HepG2 cells administered with 18-carbon FA were used. HE and Oil Red O staining were used for mice hepatic pathological examination. The mRNA and protein expressions were measured with real-time PCR and Western blot. The results showed that hepatic CYP2A5 and Nrf2 expression levels were increased in HFD fed mice accompanied with hepatic 18-carbon FA accumulation. The Nrf2 expression was increased dose-dependently in cells administered with increasing concentrations of stearic acid, oleic acid, and alpha-linolenic acid. The Nrf2 expression was dose-dependently decreased in cells treated with increasing concentrations of linoleic acid, but the Nrf2 expression level was still found higher than the control cells. The CYP2A6 expression was increased dose-dependently in increasing 18-carbon FA treated cells. The HFD-induced up-regulation of hepatic CYP2A5 *in vivo* and the 18-carbon FA treatment induced up-regulation of CYP2A6 in HepG2 cells were, respectively, inhibited by Nrf2 deficiency and Nrf2 silencing. While the basal expression of mouse hepatic CYP2A5 was not impeded by Nrf2 deletion. Nrf2 over expression improved the up-regulation of CYP2A6 induced by 18-carbon FA. As the classical target gene of Nrf2, *GSTA1* mRNA relative expression was increased in Nrf2 over expressed cells and was decreased in Nrf2 silenced cells. In presence or absence of 18-carbon FA treatment, the change of CYP2A6 expression level was similar to *GSTA1* in Nrf2 silenced or over expressed HepG2 cells. It was concluded that HFD-induced hepatic 18-carbon FA accumulation contributes to the up-regulation of CYP2A5/2A6 via activating Nrf2. However, the CYP2A5/2A6 expression does not only depend on Nrf2.

## Introduction

Non-alcoholic fatty liver disease (NAFLD) is an important chronic liver disease and metabolic syndrome, correlated with diabetes, obesity, and cardiovascular diseases (Angulo, 2002; Marchesini et al., 2003; Yoneda et al., 2012). In recent years, morbidity of NAFLD has grown not only in Eastern but also in Western countries, and high-fat intake has become an important health issue (Kojima et al., 2003; Bhala et al., 2013). A “two-hit” theory is widely advocated to explain the progression of NAFLD although the exact mechanism of NAFLD is still unknown. Lipid deposition in hepatocytes is considered to be the first hit, which caused by insulin resistance and lipometabolism disorder and resulted in non-alcoholic liver simple steatosis (SS). Oxidative stress and the imbalance of proinflammatory cytokines resulting from lipid peroxidation and the increased reactive oxygen species (ROS) are likely to be the second hit. The second hit contributes to the progressive development of non-alcoholic steatohepatitis (NASH), liver fibrosis, cirrhosis, and even hepatocellular carcinoma from SS (Bugianesi et al., 2002). Excessive fatty acids (FA) deposition and its peroxidation in hepatocytes are considered to be the major factors causing cytotoxicity and exacerbated hepatopathology. According to the reported data of our lab and others, 18-carbon FA is the most abundant in hepatic FA composition and accumulated badly in the HFD-induced liver, especially in Nrf2 deleted animals (Zhukova et al., 2014; Wang X. et al., 2016). CYP2A5 and Nrf2 expression were both induced by 18-carbon FA treatment in mouse primary hepatocytes (Cui et al., 2016). Nevertheless, the role of 18-carbon FA in inducing CYP2A6 in human hepatoma cell line has never been reported.

The metabolism and detoxification of xenobiotics including drugs and environmental toxicants mainly take place in the liver. A variety of enzymes with overlapping substrate specificity are expressed in the liver and are divided into Phase I (oxidizing) and Phase II (conjugating) drug metabolizing enzymes (DMEs). Approximately 90% of Phase I metabolism is carried out by enzymes belonging to the cytochrome P450 (CYP) superfamily. Mouse CYP2A5 and its human ortholog CYP2A6 belong to the CYP450 family. There are increasing evidence that hepatic CYP2A5 expression and activity was enhanced in the liver exposed to various chemical hepatotoxins and pathophysiological conditions [chemicals (Jounaidi et al., 1994), AFB1 (Pelkonen et al., 1997), microorganism (Sipowicz et al., 1997), carcinoma (Raunio et al., 1998), parasite (Montero et al., 1999)], while levels of most CYP enzymes are either unchanged or decreased (Kojo et al., 1998). CYP2A5/2A6 is coumarin-7-hydroxylase and responsible for the metabolism of nicotine, drugs and procarcinogens such as aflatoxin B1 (AFB1) and nitrosamines (Camus-Randon et al., 1993; Kirby et al., 1994a,b; Felicia et al., 2000). The hepatotoxic compounds that up-regulate CYP2A5/2A6 are structurally unrelated and are not considered to be CYP inducers. The mechanism of CYP2A5/2A6 increased in different pathogenesis induced liver damage is still unclear. It has been proposed that the common mechanism in the CYP2A5-inducing conditions is a direct or indirect systemic effect elicited by toxicity or tissue damage, rather than the chemical itself (Camus-Randon et al., 1996; Salonpää et al., 1997). Lipid accumulation (hepatocytes steatosis) is generally the early stage of liver damages induced by various structurally unrelated chemicals. Studies focus on the expression of hepatic CYP2A5/2A6 in hepatocytes steatosis is limited at the moment.

NF-E2-related factor 2 (NFE2L2 or Nrf2), a basic leucine zipper transcription factor that belongs to the Cap “N” Collar (CNC) family of transcription factors is expressed in diverse cell types including hepatocytes (Oyake et al., 1996). Activated by electrophiles and oxidants, Nrf2 binds to DNA sequences which named antioxidant response elements (ARE), and initiates the transcription of target genes that contribute to elimination of free radicals and electrophiles (Wakabayashi et al., 2004). In other words, Nrf2 is a key nuclear transcription factor that regulates the expression of genes against oxidative stress. Nrf2 is reported to play a cytoprotective role in NAFLD by regulating the expression of antioxidants and cytokines, thus resisting oxidation, inflammation, and fibrosis which generate the second hit of the “two-hit” theory (Chowdhry et al., 2010; Sugimoto et al., 2010; Meakin et al., 2014). Deficiency of Nrf2 in mice leads to rapid onset and progression of NAFLD. Thus, the potential of Nrf2 as the treatment target of NAFLD has been demonstrated using Nrf2 activators *in vivo* and *in vitro* (Shimozono et al., 2013).

According to former reports of our lab, *Nrf2* and *CYP2A5* mRNA expressions were both elevated in mouse model of hepatocytes steatosis (Wang C. et al., 2016) accompanied with 18-carbon FA accumulation in the hepatocytes (Wang X. et al., 2016). In mouse primary hepatocytes treated with 18-carbon FA, Nrf2, and CYP2A5 expressions were increased (Cui et al., 2016). We hypothesize that the common stimulus for up-regulation of CYP2A5/2A6 in liver damages caused by various structurally unrelated chemicals is hepatocellular FA accumulation (generally the early stage of liver damages), and Nrf2 is a potential mechanism by which 18-carbon FA induces CYP2A5/2A6 expression. Our objective is to investigate the relationship between hepatocellular 18-carbon FA accumulation and CYP2A5/2A6 expression and the involvement of Nrf2 in the process by (i) investigating the effects of hepatic steatosis on CYP2A5 expression via Nrf2 in Nrf2-null and wild type (WT) mice fed with HFD, (ii) examining the effects of 18-carbon FA [stearic acid (SA, C18:0), oleic acid (OA, C18:1), linoleic acid (LA, C18:2), and alpha-linolenic acid (ALA, C18:3)], which significantly accumulated in the HFD fed mice liver, on Nrf2 and CYP2A6 expressions in HepG2 cells, and (iii) if the effects of 18-carbon FA on CYP2A5/2A6 expression are related to Nrf2 with Nrf2 silenced or over expressed HepG2 cells. As the classical target gene of Nrf2, *GSTA1* mRNA expression was detected in Nrf2 silenced or over expressed HepG2 cells to indirectly reflect the activation of Nrf2. The results indicated that the HFD-induced hepatocellular 18-carbon FA accumulation up-regulates CYP2A5/2A6 via Nrf2 during hepatocytes steatosis. However, Nrf2 is not the only compound that regulates CYP2A5/2A6 expression.

## Materials and methods

### Animals and diets

As described in our former report (Wang X. et al., 2016), 8 week-old WT and Nrf2-null male mice with ICR background fed 8 weeks of control diet (CD) and HFD were used for experiments. All the mice were pathogen-free. Each group consisted of 10 mice. All of the experimental protocols that involve animals were approved by the Northeast Agricultural University Animal Care and Use Committee prior to the initiation of the study.

### Liver pathology

#### Paraffin processing of mice livers tissue

Liver tissue was fixed in10% neutral formalin, dehydrated in graded ethanol solutions (70, 80, 90, 95, and 100%) at 4°C and embedded in paraffin at 60°C.

#### Mouse liver pathology

Liver sections (4 μm) were dewaxed in xylene, passed through graded ethanol solutions, stained with hematoxylin and eosin (HE), and then examined by three pathologists ignorant of the mice groups and their diets.

#### Oil red O staining

Oil red O staining was performed according to the method described in our previous study (Wang X. et al., 2016). The fat accumulated in hepatocytes was demonstrated intuitively as red.

### Cell culture and treatments

HepG2 cells used in our experiments were obtained from Harbin Medical University, China. The passage number of cells used in each experiment is 3 or 4. All assays were performed with nine replicates.

#### MTT cell viability assay

1 × 10^4^ HepG2 cells were platted in 96-well plates and cultured in Dulbecco's modified eagle medium (DMEM, Gibco, NY, USA) with 10% fetal bovine serum (FBS, Invitrogen, Life Sciences, USA) and 0, 0.25, 0.5, 1, 2 millimole per liter (mM) SA, OA, LA, and ALA, respectively, for 24 h. Then, 10 μL of 5 mg/ml MTT was added into each well and 4 h incubation at 37°C was needed. Discard the supernatant. One hundred fifty microliters of dimethyl sulfoxide (DMSO, Jiancheng, Nanjing, China) was added into each well and incubated at 37°C for 10 min. Formazan in living cells was solubilized by DMSO. The absorbance was measured at 490 nm with a microplate reader (Bio-Rad iMark, USA).

#### HepG2 cells culture and treatment

HepG2 cells were cultivated in DMEM solution containing 10%FBS at 37°C in 5% CO_2_. 2 × 10^5^ HepG2 cells were platted in 6-well plate and cultivated for 6 h, and then changed the culture medium to DMEM solution without FBS. Twenty-five hours later, 0.25, 0.5, 1 mM SA, OA, LA, and ALA standards (Sigma, St. Louis, MO, USA) prepared via saponification and albumin binding were added into culture medium, respectively. A 24 h incubation was needed before harvesting the cells for Nrf2 and CYP2A6 levels detection.

#### Nrf2 gene silence in HepG2 cells

*Nrf2* siRNA was designed according to the sequence of human *Nrf2* available in GenBank. The sequences of *Nrf2* siRNA were; sense: GCUGCUCAGAAUUGCAGAAT (5′–3′), antisense: UUCUGCAAUUCUGAGCAGCTT (5′–3′). The sequences of negative control (NC) siRNA were; sense: UUCUCCGAACGUGUCACGUTT (5′–3′), antisense: ACGUGACACGUUCGGAGAATT (5′–3′). 5 × 10^4^ HepG2 cells were platted in 24-well plate. Twenty-four hours later, 15 pmol *Nrf2* siRNA and NC siRNA were, respectively, transfected into the cells with 1.5 μl Lipofectamine 2000. Twenty-four hours after transfection, replace the culture medium to DMEM solution containing 10% FBS and 1 mM LA or ALA. After incubation at 37°C for 24 h, the cells were harvested for *GSTA1* mRNA, CYP2A6 mRNA and protein detection.

#### Nrf2 gene over expression in HepG2 cells

pcDNA3-EGFP-C4-Nrf2 and pcDNA3-EGFP-C4 were the Nrf2 over expression plasmid and the NC plasmid, respectively. They were both bought from Addgene. The procedures of transfection and cells treatments are same as Section Nrf2 Gene Silence in HepG2 Cells.

### Real-time PCR

HepG2 cells were disrupted by TRIzol reagent (Invitrogen, Carlsbad, USA). The total RNA isolation and the process of reverse transcription were same as our former report (Wang X. et al., 2016). ABI 7500 sequence detection system was used in performing SYBR-Green quantitative real-time polymerase chain reaction (RT-PCR). The mRNA levels of human *CYP2A6, Nrf2*, and *GSTA1* were normalized to β*-actin*. The relative change in mRNA gene expression were calculated using the −ΔΔCt method. Primers were designed using Primer Premier5.0 software and were based on the mRNA sequences available at the National Center for Biotechnology Information (Table 1).

**Table 1:** **The primer sequences for Real-Time PCR**.

**Gene**	**Sequences of primers**
CYP2A6	F 5′-GAGGAGGAGAAGAACCCCAAC-3′
	R 5′-TGAGCAGCAAGAAGCCATAGC-3′
Nrf2 (human)	F 5′-TTCCCGGTCACATCGAGAG-3′
	R 5′-TCCTGTTGCATACCGTCTAAATC-3′
GSTA1 (human)	F 5′-GGGAAAGACATAAAGGAGAGAG-3′
	R 5′-TCAAAGGCAGGGAAGTAGC-3′
β-actin (human)	F 5′-GATCCACATCTGCTGGAAGG-3′
	R 5′-AAGTGTGACGTTGACATCCG-3′

### Western blot

Liver cells were lysed by RIPA (Beyotime, Guangzhou, China) and centrifuged at 12,000 × g for 5 min. The supernatant which contains the protein was collected. The protein in hepatocytes nucleus was extracted with the commercial Kit manufactured by Beyotime Biotechnology. The protein concentrations were determined with the BCA Assay Kit from Beyotime Biotechnology. CYP2A5, Nrf2 (mouse and human), and CYP2A6 proteins were separated by SDS-polyacrylamide gel electrophoresis (12%), electrophoretically transferred to nitrocellulose/polyvinylidene difluoride membranes (Pierce Biotechnology, Rockford, IL/Bio-Rad Laboratories, Hercules, CA), and blocked in phosphate buffer containing 0.1% Tween 20 and 5% non-fat milk for 1 h at RT. Blots were incubated with Nrf2 polyclonal antibody (rabbit anti mouse, 1:500 diluted, Bioss, Beijing, China), CYP2A5 monoclonal antibody (chicken anti mouse, 1:1000 diluted, presented by University of Oulu), CYP2A6 polyclonal antibody (rabbit anti human, 1:200 diluted, ImmunoWay Biotechnology Company), and Nrf2 polyclonal antibody (rabbit anti human, 1:200 diluted, ImmunoWay Biotechnology Company) for 1 h at 37°C. Membranes were then incubated with horseradish peroxidase-conjugated antibody (goat anti rabbit, 1:5000 diluted, Boster biological engineering co., LTD, Wuhan, China; rabbit anti chicken, 1:5000 diluted, Takara, Dalian, China) for 1 h at RT. After further washing with phosphate-buffered saline, blots were incubated in commercial chemoluminescence reagents (Amersham Biosciences, USA). Band intensities were measured using Quantity One software (BioRad, Hercules, CA). The quantity of Nrf2 protein expression in nucleus was relative to Histon H1, and in cytoplasm was relative to β-actin. Nrf2 and CYP2A5/2A6 protein expressions in hepatocytes were relative to β-actin.

### Statistical analysis

Ten replicates were used to generate an individual data point in each of the independent experiments *in vivo*. Nine replicates were used to generate an individual data point in each of the independent experiments *in vitro*. Results are expressed as the mean ± standard deviation (*SD*). A statistical software package (SPSS, version 17.0) was used for the data analyses. ANOVA was used to compare quantitative data among groups. The column charts were designed by Graphpad Prism 6.0.

## Results

### Nrf2 deletion aggravated HFD-induced hepatic steatosis

**Figure 1:**
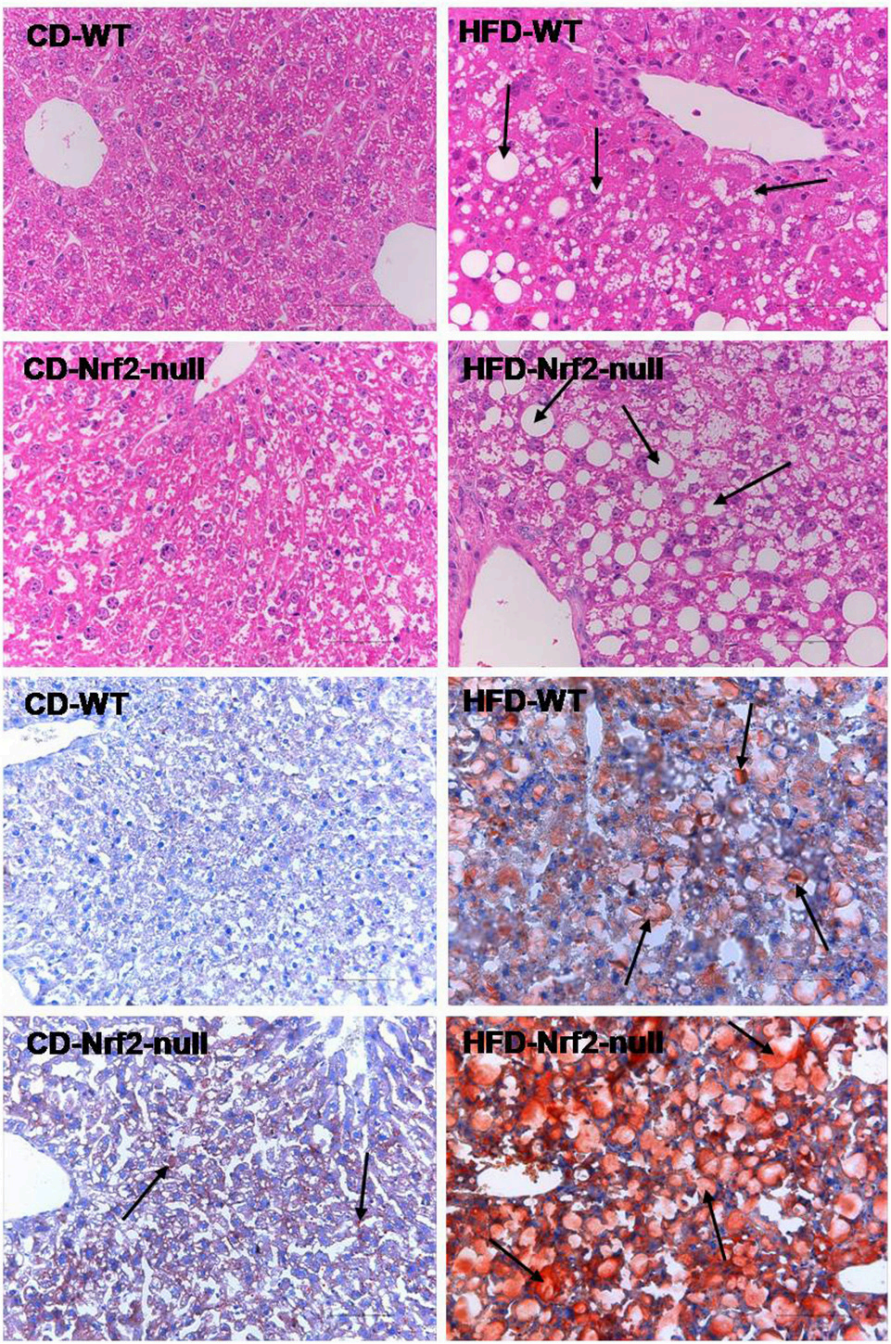
**Mouse hepatic pathology**. Liver sections of the former four figures were stained by hematoxylin and eosin. Liver sections of the latter four figures were stained with Oil Red O and the fat accumulated in hepatocytes manifested red. All the liver sections were examined by light microscopy and the images were displayed at 200× the original magnification. The fat accumulation in hepatocytes were pointed out by the arrows in HFD groups.

As shown in Figure 1, after 8 weeks of HFD feeding, livers from WT mice showed micro- and macro-vesicular fat accumulation. In contrast, livers from Nrf2-null mice were more sensitive to HFD feeding than WT mice with evidence of greater macrovesicular fat accumulation. In addition, the Oil Red O staining mice liver sections showed that fat accumulated much more in HFD-Nrf2-null mice hepatocytes than HFD-WT mice. In the liver of CD-Nrf2-null mice, there was slight hepatocellular fat accumulation. However, there is no evidence of fat accumulation in the CD-WT mice liver. In addition, data in Wang X. et al., (2016) also gives evidence of FA accumulation in the liver of HFD fed mice. Nrf2 deficiency improved the hepatic FA accumulation induced by HFD, especially the 18-carbon FA. As the predominant components of hepatic FA, 18-carbon FA accumulated significantly in HFD fed mice liver of both genotypes (WT and Nrf2-null mice).

### Nrf2 deletion inhibited HFD-induced mouse hepatic CYP2A5 expression

**Figure 2:**
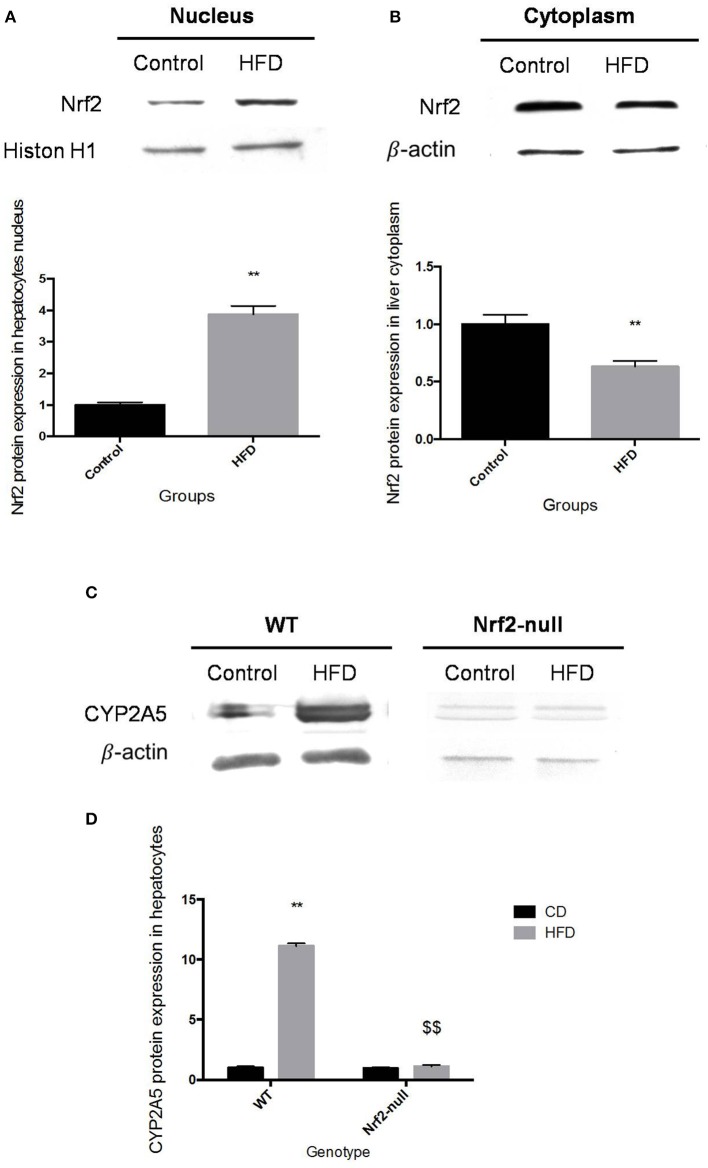
**Nrf2 and CYP2A5 protein expressions in HFD feeding mice**. Nrf2 protein expression in mouse hepatocytes nucleus and its quantity relative to Histon H1 is shown in panel **(A)**. Nrf2 protein expression in liver cytoplasm and its quantity relative to β-actin is shown in panel **(B)**. Hepatic CYP2A5 protein expression in WT and Nrf2-null mice, which fed with CD and HFD, is revealed in panel **(C)** and the quantity relative to β-actin is shown in panel **(D)**. ^*^ represent statistical difference caused by HFD within WT or Nrf2-null groups; $ represent statistical difference caused by Nrf2 deficiency on the same diet. ^*^ 0.01 < *P* < 0.05, ^**^P < 0.01; $ 0.01 < *P* < 0.05, $$*P* < 0.01.

In HFD-WT mice, the Nrf2 protein expression level in hepatocytes nucleus was multiplied by 3.87 in comparison with the control group (Figure 2A). While in liver cytoplasm the Nrf2 protein expression level was decreased by 36.8% in HFD-WT mice compared to the control (Figure 2B). The hepatic CYP2A5 protein expression level was multiplied by 11.13 in HFD-WT mice compared to CD-WT mice (Figures 2C,D). However, in Nrf2-null mice the expression level of CYP2A5 in hepatocytes was not changed by HFD feeding (Figures 2C,D). In the control groups, Nrf2 deficiency did not influence the basal expression of hepatic CYP2A5.

### Nrf2 and CYP2A6 expression levels were increased in HepG2 cells treated with 18-carbon FA

**Figure 3:**
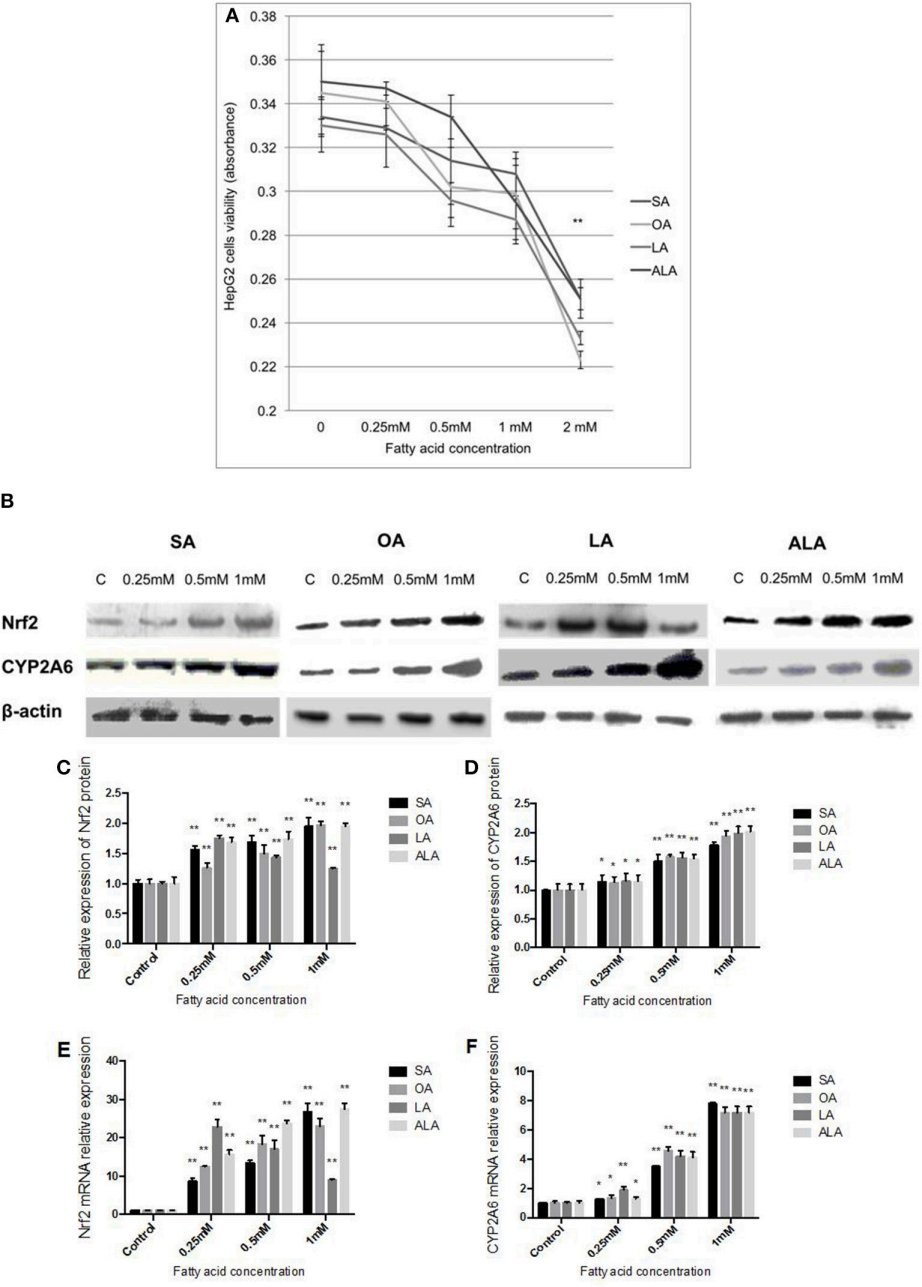
**Effect of 18-carbon fatty acid on Nrf2 and CYP2A6 expression in HepG2 cells. (A)** HepG2 cells viability which were administered with increasing concentrations of SA, OA, LA, and ALA. The cells viability was represented by the absorbance. The effect of SA, OA, LA, and ALA on Nrf2 and CYP2A6 protein expression in HepG2 cells are exhibited in panel **(B)**. Nrf2 protein expression was quantified and shown in panel **(C)**. CYP2A6 protein expression was quantified and shown in panel **(D)**. The effect of SA, OA, LA, and ALA on Nrf2 and CYP2A6 mRNA expressions are shown in panel **(E,F)**, respectively. Asterisks ^*^ represent statistical difference caused by fatty acid administration from control cells. ^*^ 0.01< *P* < 0.05, ^**^P < 0.01.

The low concentrations (0.25, 0.5, 1 mM) of SA, OA, LA, and ALA have no significant effect on the viability of HepG2 cells. However, HepG2 cells viability was significantly inhibited (over 25%) as the concentration of FA was 2 mM (Figure 3A). Nrf2 protein (Figures 3B,C) and mRNA (Figure 3E) expression was increased with the increasing doses of SA, OA, and ALA (0.25, 0.5, 1 mM). Nrf2 protein (Figures 3B,C) and mRNA (Figure 3E) expression was decreased gradually in HepG2 cells administered with increasing doses of LA (0.25, 0.5, 1 mM), but these expression levels were found higher than the control group. CYP2A6 protein (Figures 3B,D) and mRNA (Figure 3F) expressions were both dose-dependently up-regulated in HepG2 cells treated with SA, OA, LA, and ALA in comparison with the control cells.

### Nrf2 silence inhibited 18-carbon FA induced CYP2A6 expression, Nrf2 over-expression accelerated CYP2A6 expression

**Figure 4:**
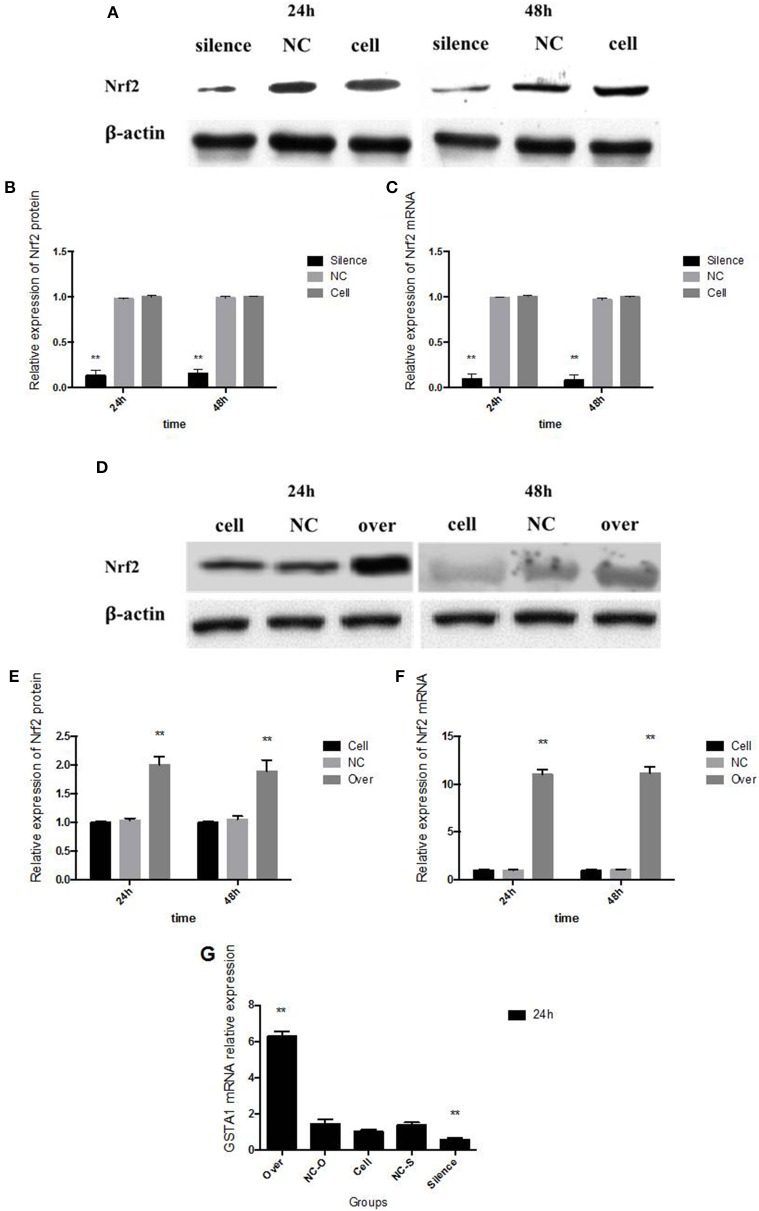
**Nrf2 gene silence and over expression**. In Nrf2 siRNA transfected HepG2 cells, Nrf2 protein expression is shown in panel **(A)** and its quantity relative to β-actin is shown in panel **(B)**; Nrf2 mRNA expression is shown in panel **(C)**. In pcDNA3-EGFP-C4-Nrf2 transfected HepG2 cells, Nrf2 protein expression is shown in panel **(D)** and its quantity relative to β-actin is shown in panel **(E)**; Nrf2 mRNA expression is shown in panel **(F)**. The *GSTA1* mRNA relative expression in different groups was shown in panel **(G)**. NC, in panel **(A–C)** represents the HepG2 cells that transfected with negative control siRNA, in panel **(D–F)** represents the HepG2 cells that transfected with negative control plasmid (pcDNA3-EGFP-C4). In panel **(G)**, NC-O represents the cells that transfected with pcDNA3-EGFP-C4 and NC-S represents the HepG2 cells that transfected with negative control siRNA. Cell, means the control cells that without any administration. Asterisks ^*^ represent statistical difference caused by Nrf2 siRNA or pcDNA3-EGFP-C4-Nrf2 transfection from control cells without any stimulation. ^*^ 0.01< *P* < 0.05, ^**^P < 0.01.

**Figure 5:**
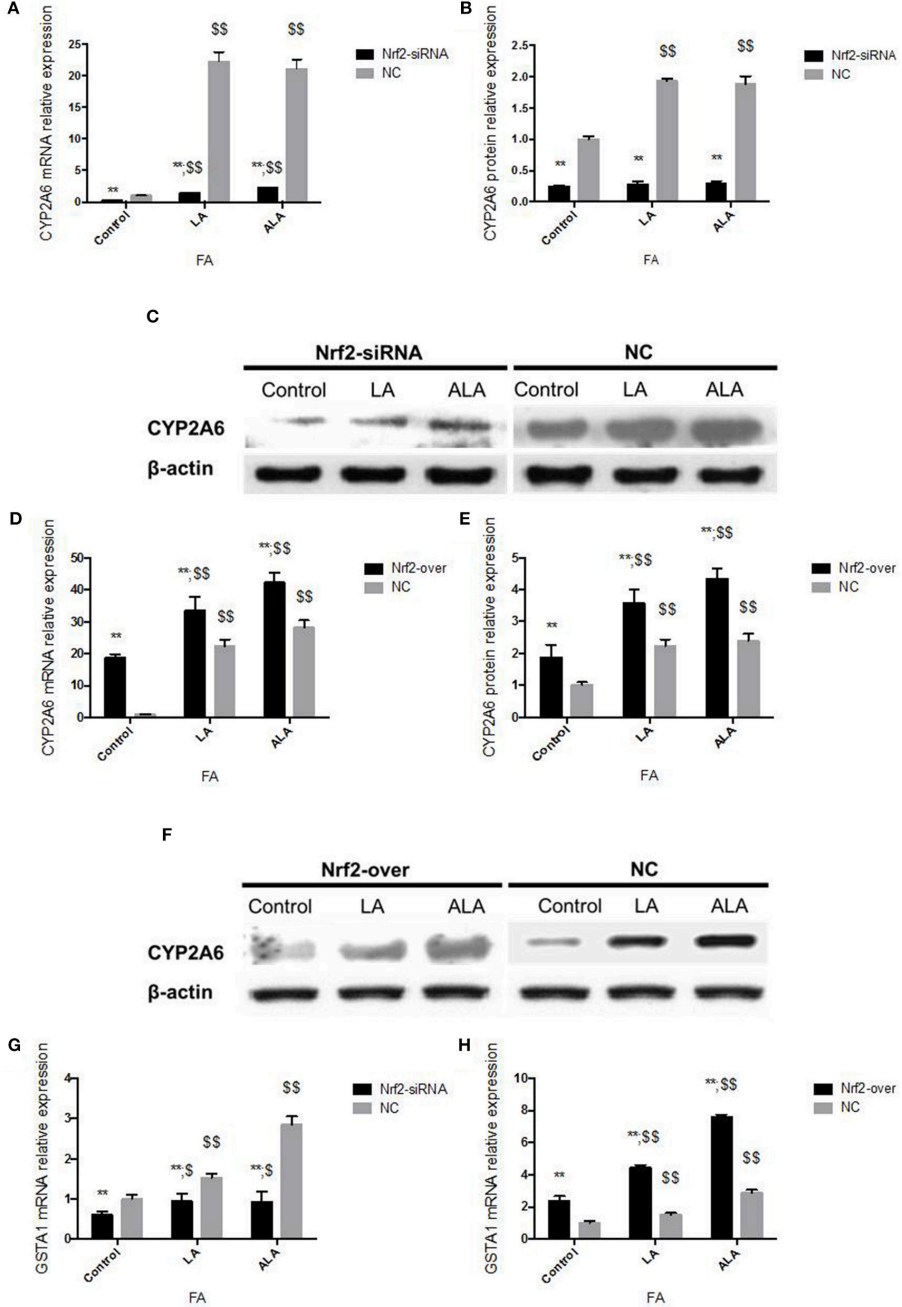
**CYP2A6 and GSTA1 expression changes in Nrf2 silenced and over-expressed HepG2 cells administered with LA and ALA**. CYP2A6 mRNA **(A)** and protein **(B,C)** expressions were both decreased significantly in Nrf2 silenced cells with or without LA and ALA treatment. CYP2A6 mRNA **(D)** and protein **(E,F)** expressions were increased in Nrf2 over-expressed cells with or without LA and ALA administration. In the presence or absence of LA and ALA administration, *GSTA1* mRNA expression was down-regulated in cells transfected with Nrf2 siRNA **(G)** and was up-regulated in cells transfected with pcDNA3-EGFP-C4-Nrf2 **(H)**. In addition, the NC cells in panel **(A–C,G)** were transfected with NC siRNA. The NC cells in panel **(D–F,H)** were transfected with NC plasmid (pcDNA3-EGFP-C4). ^*^ represent statistical difference caused by transfection of Nrf2 siRNA and pcDNA3-EGFP-C4-Nrf2 from cells transfected with NC siRNA and NC plasmid; $ represent statistical difference caused by LA and ALA administration within Nrf2 silenced cells, Nrf2 over expressed cells, and control cells. ^*^0.01 < *P* < 0.05, ^**^P < 0.01; $ 0.01 < *P* < 0.05, $$*P* < 0.01.

Transfected with *Nrf2* siRNA, Nrf2 protein expression declined 86% in 24 h and 84% in 48 h, respectively (Figures 4A,B). Transcription of *Nrf2* in HepG2 cells declined 90.10% in 24 h and 91.26% in 48 h, respectively (Figure 4C). Twenty-four hours after pcDNA3-EGFP-C4-Nrf2 was transfected in HepG2 cells, Nrf2 protein (Figures 4D,E), and mRNA (Figure 4F) expression was multiplied by 2.01 and 10.88, respectively. Forty-eight hours after pcDNA3-EGFP-C4-Nrf2 was transfected, Nrf2 protein (Figures 4D,E) and mRNA (Figure 4F) expression was multiplied by 1.89 and 10.84, respectively. *GSTA1* mRNA relative expression showed an elevation of 528% in cells transfected with pcDNA3-EGFP-C4-Nrf2 and was decreased by 43.1% in cells transfected with *Nrf2* siRNA (Figure 4G). In cells transfected with NC plasmids (NC siRNA or pcDNA3-EGFP-C4), Nrf2, and *GSTA1* expressions showed no statistical difference compared to the control cells (Figure 4). CYP2A6 mRNA and protein expressions were increased by LA and ALA treatment in HepG2 cells transfected with functional plasmids or NC plasmids. HepG2 cells were examined for mRNA and protein expression levels in the presence or absence of LA and ALA treatment, the expressions of mRNA (Figure 5A) and protein (Figures 5B,C) levels of CYP2A6 were extremely reduced in Nrf2 silenced cells compared to the NC groups. Conversely, Nrf2 over-expressed HepG2 cells expressed much higher CYP2A6 mRNA (Figure 5D) and protein (Figures 5E,F) levels than the cells transfected with NC plasmid. In the presence or absence of LA and ALA treatment, *GSTA1* mRNA relative expression was reduced in Nrf2 silenced cells (Figure 5G) and was increased in Nrf2 over expressed cells (Figure 5H) compared to the NC groups.

## Discussion and conclusions

Hepatic CYP2A5/2A6 is reported up-regulated in various pathophysiological liver damages and induced by structurally variable hepatotoxic chemicals (Jounaidi et al., 1994; Pelkonen et al., 1997; Sipowicz et al., 1997; Raunio et al., 1998; Montero et al., 1999), including NAFLD (Wang C. et al., 2016). In all of these conditions, lipid accumulation in hepatocytes always occurs at the early stage of liver damage and results in redox status disorder in hepatocytes, which contributes to the second hit. Nrf2 is a transcription factor that activated by oxidative stress and regulates the transcription of numerous cytoprotective target genes. Our former researches showed that hepatic *Nrf2* and *CYP2A5* mRNA expressions were increased significantly in HFD fed mice (Wang C. et al., 2016) and accompanied with badly hepatic 18-carbon FA (SA, OA, LA, and ALA) accumulation, which is the most abundant component of liver FA (Wang X. et al., 2016). Meanwhile, mice primary hepatocytes treated with 18-carbon FA also express more Nrf2 and CYP2A5 than the control cells (Cui et al., 2016). Thus, we hypothesize that HFD-induced hepatic 18-carbon FA accumulation up-regulates CYP2A5 in hepatocytes steatosis may correlate with the activation of Nrf2. Whether Nrf2 expression is necessary for CYP2A5/2A6 up-regulation in hepatocellular steatosis has never been reported. Our study aims to investigate the necessity of Nrf2 expression for hepatic 18-carbon FA accumulation induced CYP2A5/2A6 up-regulation.

Serological (Wang X. et al., 2016) and pathological tests indicated that mice developed liver steatosis that was exacerbated by Nrf2 deletion after 8 weeks of HFD feeding. In condition of mild damage in hepatocytes, ALT from the cytoplasm leaks out because the membrane permeability is enhanced. While in condition of severe damage in hepatocytes, AST from mitochondria leaks out due to the disruption of mitochondrial membrane. Compared to the HFD-WT mice, the increase of AST and the decrease of ALT in HFD-Nrf2-null mice indicated that Nrf2 deficiency enhanced the hepatocytes damage induced by HFD feeding. ALP leaks into blood from hepatocytes in condition of liver damage. Kidney is considered to be the major organ that produces GGT, but the GGT in serum primarily comes from the hepatobiliary system. In heavily injured hepatocytes, GGT existed in the smooth endoplasmic reticulum will leak out into blood. Thus, the increase of serum ALP and GGT levels induced by Nrf2 deletion in HFD fed groups also demonstrated that Nrf2 deficiency accelerated the HFD-induced liver damage. Moreover, the grown macrovesicular fat accumulation in HFD-Nrf2-null mice liver in comparison with the HFD-WT mice indicated that Nrf2 deficiency intensified the liver sensibility to HFD feeding.

The changes of CYP2A5 protein expression and Nrf2 nuclear translocation showed the same trend in HFD fed WT mice liver compared to the control mice (Figure 2). Considering the hepatic FA accumulation (Wang X. et al., 2016), the increase of CYP2A5 protein expression and Nrf2 nuclear translocation in HFD-WT mice demonstrated that HFD-induced FA accumulation up-regulated CYP2A5 and activated Nrf2. However, the hepatic CYP2A5 protein expression in HFD-Nrf2-null mice approached to the basal level in mice fed with CD. This phenomenon indicated that expression of Nrf2 is crucial for the up-regulation of mouse hepatic CYP2A5 induced by HFD feeding. Another research based on mice exposed to cadmium chloride (16 mmol/kg body weight) reported that cadmium alters cellular redox status, induced hepatic CYP2A5 in WT mice but not in Nrf2-null mice (Abu-Bakar et al., 2004). Our study is in agreement with the above finding that Nrf2 may be the common regulator which contributes to the up-regulation of CYP2A5 in liver injuries induced by structurally irrelevant pathogenesis. Our data also showed that in Nrf2-null mice, the increase of CYP2A5 induced by hepatic FA accumulation was inhibited but the hepatocellular damage was enhanced which indicated that CYP2A5 up-regulation does not contribute to the development of the HFD-induced liver damage.

As shown in Wang X. et al. (2016), the 18-carbon FA showed high concentration (almost 60%) in mouse liver and high sensitivity to Nrf2 deficiency, which indicated that 18-carbon FA is the predominant component contributed to the formation of hepatocytes steatosis. Thus, SA (C18:0), OA (C18:1), LA (C18:2), and ALA (C18:3) were chosen as the HepG2 cells stimulus to investigate the role of 18-carbon FA in inducing CYP2A6 and the necessity of Nrf2 in the process. HepG2 cell line was used in this study because it regenerates easily and very similar to human normal hepatocytes in resisting lipid accumulation (Chao et al., 2010; Chavez-Tapia et al., 2011; Yao et al., 2011).

FA showed strong cytotoxicity via inhibiting the cell growth and even causing cell death at certain concentrations. Toxicity of FA on cells depends on the concentration, solvent, cell type and culture condition, which lead to different FA concentrations that were used in different studies. In our study, the FA that used in stimulating HepG2 cells was prepared via saponification and albumin binding, so that the toxicity of DMSO or ethanol on cell viability was avoided. MTT cell viability assay revealed that the optimal FA concentrations for inducing HepG2 cells steatosis without significant inhibition in cell viability were 0.25, 0.5, and 1 mM (Figure 3A). Nrf2 and CYP2A6 expression induced by 18-carbon FA (SA, OA, LA, and ALA) *in vitro* (Figures 3B–F) showed the same tendency as the experiments *in vivo* (Figure 2), which indicated that the experiment *in vitro* reproduced the process *in vivo* to a certain extent. Interestingly, Nrf2 mRNA and protein expressions were dose-dependently increased in HepG2 cells exposed to SA, OA, and ALA, but dose-dependently decreased in HepG2 cells exposed to LA, although still much higher than the control cells. The current reports showed that although LA and its metabolite derivatives play cytoprotective role in HepG2 cells and mouse/rat organs via inducing the expression of Nrf2 and its downstream antioxidative genes (Mollica et al., 2014; Furumoto et al., 2016), LA concentrations higher or lower than the optimal value attenuated its auxo-action on Nrf2 expression (Zeng et al., 2016). However, the exact mechanism of diverse LA concentrations (with similar cytotoxicity) showed different effects on inducing Nrf2 expression remains to be investigated.

Nrf2 or Keap1 activators, inhibitors and primary hepatocytes of WT and Nrf2-null mice have been used in some studies focused on the relationship between Nrf2 and CYP2A5 in liver (Abu-Bakar et al., 2004; Lämsä et al., 2010; Shimozono et al., 2013). Furthermore, the expression of Nrf2 can't be inhibited by inhibitors fundamentally (Jnoff et al., 2014), and the primary hepatocytes continuous passage hard. In our experiment, Nrf2 silenced and over-expressed cell models were established successfully by transfected with Nrf2 specific siRNA and pcDNA3-EGFP-C4-Nrf2 in HepG2 cells using liposome-mediated method. The eukaryotic expression vector or siRNA combined with liposome and got into cells by endocytosis to accomplish Nrf2 over expression or silencing transiently. It was observed from Figure 4 that the transfection did not showed statistically significant effect on the cell viability at both time points (24 and 48 h), and Nrf2 mRNA & protein expressions in the Nrf2 silenced and over expressed cell models were found to be declined or improved. Twenty-four hours of time-point was chosen in the current study because longer liposome effect on cells causes heavier hepatocytes damage, and the Nrf2 silenced and over expressed results almost the same in 24 and 48 h. The variation trend of *GSTA1* mRNA relative expression was similar as Nrf2 in Figure 4. This result indirectly indicated that Nrf2 gene silencing and over expression in Nrf2 silenced and over-expressed cell models were satisfactorily used.

It is well-known that LA and ALA are important precursors of many long chain FAs *in vivo*. LA and ALA cannot be synthesized by the body or cells and must be absorbed from food. Our data showed that LA and ALA at 1 mM were the most effective activator of CYP2A6. Thus, LA and ALA were chosen as the stimulus of Nrf2 silenced and over expressed HepG2 cells in the current study to scrutinize the involvement of Nrf2 in regulating CYP2A6. As shown in Figure 5, the expression of CYP2A6 induced by LA and ALA was significantly attenuated in Nrf2 silenced cells, and was markedly enhanced in Nrf2 over expressed cells, which indicated that Nrf2 expression was very crucial for the LA and ALA induced CYP2A6 up-regulation. As the classical target gene of Nrf2, *GSTA1* mRNA expression change in this experiment was similar to CYP2A6, which also indirectly proved that CYP2A6 expression was influenced by Nrf2. Is the gene of *CYP2A5/2A6* located on a chromosome regulated by Nrf2? Whether Nrf2 is the sole compound regulates CYP2A6 in hepatocytes steatosis? Two putative stress response elements (StRE) within the promoter of mouse Cyp2a5 at positions −2514 to −2505 and −2386 to −2377 have been identified with computer-based sequence analysis, which may interact with Nrf2 (Abu-Bakar et al., 2007). In our study, the expression of Nrf2 and CYP2A6 showed inverse tendency in cells treated with increasing concentrations of LA, which indicated that Nrf2 was not the only pathway contributes to the increase of CYP2A6 induced by hepatic 18-carbon FA accumulation. An aryl hydrocarbon receptor (AHR)-dependent pathway has been reported to associate with the up-regulation of CYP2A5 and a putative AHR response element (XRE) was identified in the Cyp2a5 promoter at the position −2514 to −2492 using luciferase reporter gene assays (Arpiainen et al., 2005). In addition, the dexamethasone (DEX) induced CYP2A6 increase in human primary hepatocytes was attenuated by the glucocorticoid receptor (GR) antagonist, and a mutation of hepatic nuclear factor 4 (HNF4) alpha response element (HNF4-RE), which suggested that GR and HNF4 alpha involved in the induction of CYP2A6 by DEX (Onica et al., 2008). However, how many genes contribute to the up-regulation of CYP2A5/2A6 induced by hepatocytes steatosis remains to be investigated.

In conclusion, our data give us a clue that HFD-induced hepatic 18-carbon FA accumulation up-regulates CYP2A5/2A6 via Nrf2 during hepatocellular steatosis. However, Nrf2 is not the only molecule that regulates the expression of CYP2A5/2A6.

## Author contributions

XheW and XZ designed this study and contributed to the paper writing. XC, XS, and XhuiW established Nrf2 silenced and over expressed HepG2 cell models, and participated in western blot assay and the paper writing. XL and YQ participated in the HepG2 cells viability assay and animal feeding. MH, WL, and IM conducted the experiment of liver pathology and real-time PCR assay.

### Conflict of interest statement

The authors declare that the research was conducted in the absence of any commercial or financial relationships that could be construed as a potential conflict of interest. The reviewer MB and handling Editor declared their shared affiliation, and the handling Editor states that the process nevertheless met the standards of a fair and objective review.

## Abbreviations

ALA: α-linolenic acid
CD: control diet
FA: fatty acids
GGT: gamma-glutamyltransferase
HFD: high fat diet
LA: linoleic acid
Nrf2: NF-E2-related factor 2
OA: oleic acid
SA: stearic acid
SS: simple steatosis
TFA: total fatty acids.
